# Cationic liposomes bearing Bet v 1 by coiled coil-formation are hypo-allergenic and induce strong immunogenicity in mice

**DOI:** 10.3389/falgy.2022.1092262

**Published:** 2023-01-10

**Authors:** Hans Warmenhoven, Romain Leboux, Athanasios Bethanis, Jolinde van Strien, Adrian Logiantara, Hans van Schijndel, Lorenz Aglas, Leonie van Rijt, Bram Slütter, Alexander Kros, Wim Jiskoot, Ronald van Ree

**Affiliations:** ^1^ Department of Experimental Immunology, Amsterdam University Medical Centers, Location AMC, Amsterdam, Netherlands; ^2^ HAL Allergy BV, J.H. Oortweg, Leiden, Netherlands; ^3^ Division of BioTherapeutics, Leiden Academic Centre for Drug Research, Leiden University, Leiden, Netherlands; ^4^ Department of Biosciences, University of Salzburg, Salzburg, Austria; ^5^ Department of Supramolecular & Biomaterials Chemistry, Leiden Institute of Chemistry, Leiden University, Leiden, Netherlands; ^6^ Department of Otorhinolaryngology, Amsterdam University Medical Centers, Location AMC, Amsterdam, Netherlands

**Keywords:** aluminum, bet v 1, hypo-allergenic, liposomes, mice

## Abstract

Although aluminum hydroxide (alum) is widely accepted and used as safe vaccine adjuvant, there is some concern about possible toxicity upon long-lasting repeated exposure during subcutaneous allergen immunotherapy (SCIT). Our objective was to evaluate allergen-bearing liposomes as possible alternative for alum-adsorption in SCIT. A self-assembling, coiled-coil forming peptide pair was used to anchor the major birch pollen allergen Bet v 1 to the surface of cationic liposomes. The resulting nanoparticulate liposomes were characterized with respect to their physicochemical, allergenic and immunological properties. Allergenicity was studied by ImmunoCAP inhibition and rat basophil leukemia (RBL) cell assays. Immunogenicity (immunoglobulin responses) and immune skewing (cytokine responses) were evaluated upon immunization of naïve mice, and compared to alum-adsorbed Bet v 1. Bet v 1-bearing cationic liposomes with a diameter of ∼200 nm showed a positive zeta potential. The coiled-coil conjugation of Bet v 1 to the surface of liposomes resulted in about a 15-fold lower allergenicity than soluble Bet v 1 as judged by RBL assays. Moreover, the nanoparticles induced Bet v 1-specific IgG_1_/IgG_2a_ responses in mice that were several orders of magnitude higher than those induced by alum-adsorbed Bet v 1. This strong humoral response was accompanied by a relatively strong IL-10 induction upon PBMC stimulation with Bet v 1. In conclusion, their hypo-allergenic properties, combined with their capacity to induce a strong humoral immune response and a relatively strong IL-10 production, makes these allergen-covered cationic liposomes a promising alternative for aluminum salt-adsorption of allergen currently used in SCIT.

## Introduction

Subcutaneous allergen immunotherapy (SCIT) has been used to treat allergies for more than 100 years (1). The treatment commonly consists of monthly subcutaneous injections of allergen extracts for 3 to 5 years to achieve optimal therapeutic effect. Therapy adherence is relatively low because of this long duration and the allergic side-effects that can occur (2). Often, aluminum hydroxide (alum) or aluminum phosphate is used as adjuvant for SCIT. Although alum has been reported to skew towards T helper (Th) 2 immune responses (3), during SCIT it has been shown to result in a more mixed Th1/Th2 cytokine response in combination with production of interleukin (IL)-10 by regulatory T- and B-cells (4, 5). Most importantly, these regulatory B-cells also produce the required protective allergen-specific immunoglobulin (Ig) G_4_ antibodies.

Alum has a long history of safe use in vaccines for infectious diseases but also in SCIT (3). Nevertheless, there is some concern with respect to long-term exposure to alum during allergen immunotherapy (AIT), particularly in a pediatric setting (6). Although as yet there is little or no evidence to support aluminum-associated pathology during AIT, a search for good alternatives may nevertheless be warranted. Besides directing the immune response, alum also serves as a depot for adsorption of allergens, partially shielding them from IgE antibodies and thereby reducing the risk of allergic side-effects (7). In recent years, different types of nanoparticles have drawn attention to serve as effective vaccine delivery systems (8–10). Liposomes are amongst the most promising nanoparticles to replace alum (11, 12).

Liposomes consist of one or more lipid bilayers with an aqueous core and are a versatile delivery system and adjuvant for vaccines (11–13). Antigens can be adsorbed to the lipid bilayer (14), incorporated in the lipid bilayer (15), or encapsulated in the aqueous core of the vesicle (16, 17). Recently, we described a novel antigen-anchoring method based on the interaction between two complementary *α*-helical peptides that form a coiled-coil (CC) structure (18). Immunization of mice with antigen attached to cationic liposomes *via* CC formation resulted in strong CD4^+^ T-cell proliferation and production of both interferon gamma (IFN-*γ*) and IL-10 (18). These cytokines are Th1 and regulatory T-cell responses signatures, respectively, and both are reported to be required for effective allergen immunotherapy (AIT) (8, 19–21).

The goal of this study was to design an alum-free candidate for SCIT using Bet v 1, the major allergen in birch pollen allergy, and liposomes. We produced a fusion protein between Bet v 1 and one of the two CC-forming peptides, peptide E (pepE-Bet v 1), to anchor the allergen to cationic liposomes carrying the complimentary CC-forming peptide K (pepK) at its surface. The resulting liposome nanoparticles were characterized with regard to their physicochemical, allergenic and immunological properties.

## Materials and methods

### Chemicals and reagents

Cholesterol, 1,2-distearoyl-*sn*-glycero-3-phosphocoline (DSPC) and 1,2-dioleoyl-3-trimethylammonium-propane (DOTAP) were purchased from Avanti Lipids (Birmingham, AL, United States). Recombinant Bet v 1 (isoform Bet v 1.0101, hereafter called Bet v 1) was produced by the Department of Molecular Biology of the University of Salzburg (Salzburg, Austria) (22). Dimethylformamide (DMF), piperidine, acetic anhydride, pyridine, trifluoroacetic acid (TFA) and acetonitrile (ACN) were purchased from Biosolve (Valkenswaard, The Netherlands). N,N-diisopropylethylamine (DIPEA), and ethyl cyanohydroxyiminoacetate (Oxyma) were obtained from Carl Roth (Karlsruhe, Germany). Dicholoromethane (DCM) and diethyl ether were supplied by Honeywell (Landsmeer, The Netherlands). Tentagel HL-RAM was obtained from Rapp Polymere (Tübingen, Germany). All amino acids were supplied by NovaBioChem (Darmstadt, Germany). Fmoc-NH-PEG_4_-COOH was purchased from Iris Biotech GmbH (Marktredwitz, Germany). Pierce BCA assay, Imject ® Alum and Fetal calf serum (FCS) were purchased from Thermo Fisher Scientific (Waltham, MA, United States). Isopropyl *β*-D-1-thiogalactopyranoside was obtained from Invitrogen (Carlsbad, CA, United States). Sucrose, 4-(2-hydroxyethyl)-1-piperazineethanesulfonic acid (HEPES), Triisopropylsilane (TIPS), sodium azide, Tyrodés salts, BSA, lysozyme, sodium bicarbonate, 4-methyl umbelliferyl-N-acetyl-beta-D-glucosaminide, hexafluorophosphate azabenzotriazole tetramethyl uranium (HATU), Triton X-100 and 3-(4,5-dimethylthiazol-2-yl)-2,5-diphenyltetrazolium bromide (MTT) were obtained from Sigma-Aldrich (St. Louis, MO, United States). Disodium hydrogen phosphate and sodium dihydrogen phosphate were purchased from Merck (Darmstadt, Germany). Ampicillin was obtained from Roche (Basel, Switzerland).

### Mice

Six to eight weeks old female BALB/c mice were purchased from ENVIGO (Venray, The Netherlands). The animals were housed under specific pathogen-free conditions at the animal facility of the Amsterdam University Medical Centers, location AMC. All experiments were performed in compliance with the Dutch government guidelines and the Directive 2010/63/EU of the European Parliament and were approved by the Animal Ethics Committee of the AMC.

### Peptide synthesis

Triple and quadruple repeats of the KIAALKE amino acid sequence (pepK) were synthesized by standard Fmoc chemistry using solid-phase peptide synthesis with an automated microwave peptide synthesizer (CEM liberty blue). Peptide K_3_, CGWG-(KIAALKE)_3_, was synthesized for affinity purification. Peptide K_4,_ (KIAALKE)_4_, was conjugated to cholesterol (forming CPK) *via* a poly(ethylene glycol) spacer to enable anchoring of the peptide into the lipid bilayer of liposomes and was synthesized as described elsewhere (18). In short, Fmoc-NH-PEG_4_-COOH was coupled to resin-bound (KIAALKE)_4_ in the presence of DIPEA (5 eq.) and HATU (2.5 eq.) for 2.5 h. Fmoc-deprotection was done with 20% piperidine in DMF and the reactive amine was coupled to amino-cholestene hemi-succinate (1.05 eq.) in the presence of DIPEA (5 eq.) and HATU (2.5 eq.) for 4 h at room temperature. The peptide was cleaved from the resin with a mixture of TFA:TIPS:water (95:2.5:2.5 v/v/v) and precipitated in ice-cold diethyl ether.

Crude peptides were purified using a Shimadzu RP-HPLC system comprising two LC-8A pumps and an SPD-10AVP UV-Vis detector equipped with a Kinetic Evo C18 column. A gradient of 20%–80% B, (where B is 1% (v/v) TFA in ACN, and A is 1% (v/v) TFA in water) with a flow rate of 12 ml/min was used. Collected fractions were measured on a LC-MS system (Thermo Scientific TSQ quantum access MAX mass detector connected to an Ultimate 3,000 liquid chromatography system fitted with a 50 × 4.6 mm Phenomenex Gemini 3 *μ*m C18 column). The resulting chromatogram and spectrum are shown in Supplementary Figure S1. ACN was removed by rotary evaporation (150 mbar, 50 °C) before lyophilization and the purified peptide was stored at −20 °C until use.

### Design, expression and purification of pepE-bet v 1

PepE, (EIAALEK)_3_, the complementary peptide of the pepE/K self-assembling peptide pair was attached to the N-terminus of Bet v 1. A detailed description of pepE-Bet v1 design is given in the supplementary Materials and Methods. In short, the pepE-Bet v 1 gene was used to transfect *E. coli* BL21 (DE3) cells for protein production. The harvested cell pellets were disrupted by sonication and cellular debris was removed by centrifugation. The pepE-Bet v 1 protein was purified from the supernatant by affinity purification using cross-linked agarose beads functionalized with PepK. Bound pepE-Bet v 1 was eluted by lowering the pH to 2.5 to unfold the pepE/pepK coiled coil. Elution fractions containing pepE-Bet v 1 were pooled and loaded onto a Superdex 75 pg column for polishing. Pure protein fractions were pooled and stored at −20 °C until further use. The protein concentrations of pepE-Bet v 1 and Bet v 1 were determined by BCA assay using BSA as a standard. The secondary structure of the protein was measured with circular dichroism spectroscopy (CD) and is described in detail in the supplements.

### Preparation of liposomes

**Figure 1:**
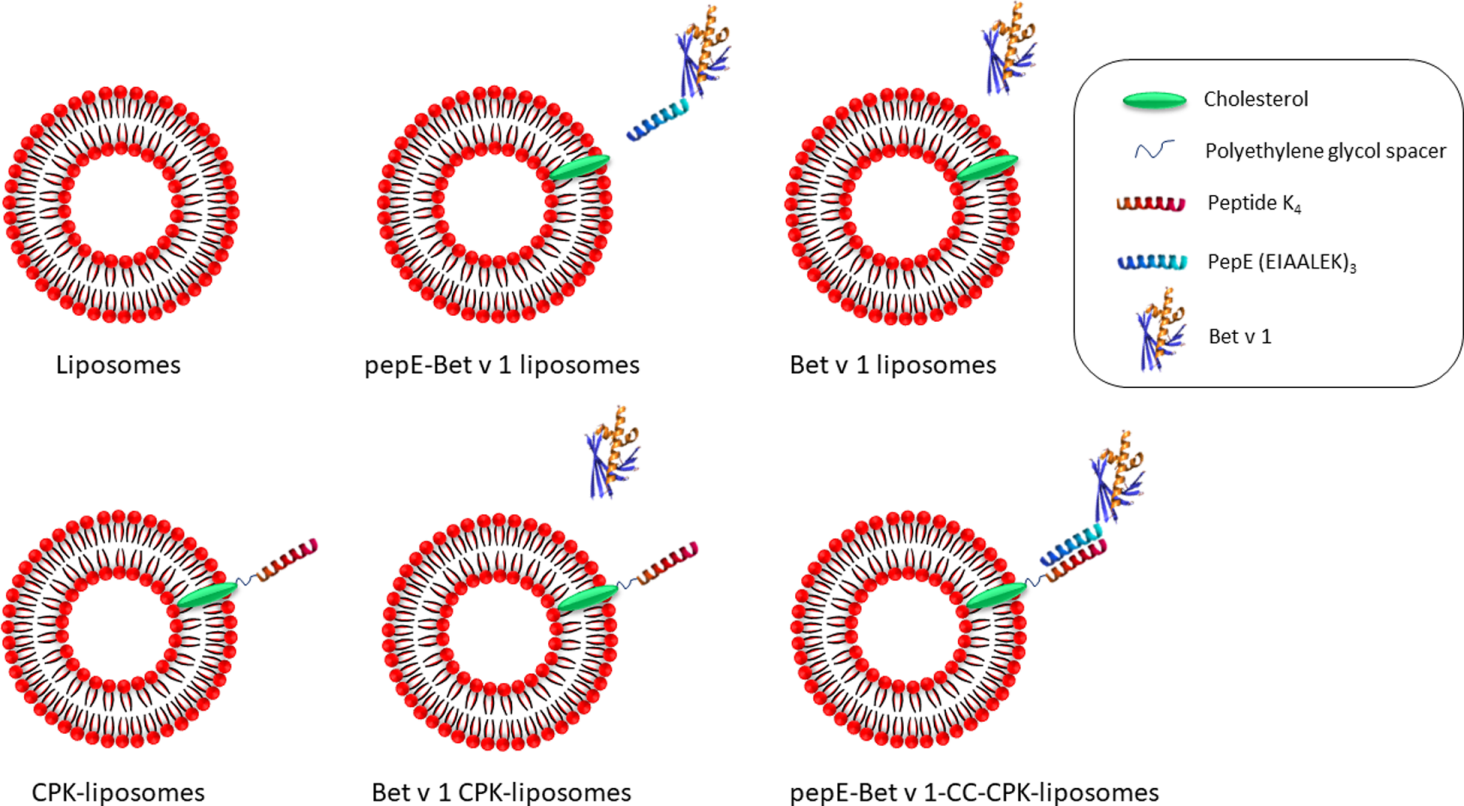
Schematic design of the liposomes used in this study. DSPC, DOTAP and cholesterol were mixed to generate liposomes (top left). The liposomes were functionalized with peptide K_4_ attached to a cholesterol anchor in the lipid bilayer *via* a polyethylene glycol spacer (CPK-liposomes, bottom left). PepE fused to the N-terminus of Bet v 1 enabled coiled coil mediated attachment to the CPK-liposomes (pepE-Bet v 1-CC-CPK-liposomes, bottom right). As control groups, pepE-Bet v 1 was added to liposomes (pepE-Bet v 1 liposomes, top middle), Bet v 1 was added to liposomes (Bet v 1 liposomes, top right) or Bet v 1 was added to CPK-liposomes (Bet v 1 CPK-liposomes, bottom middle).

CPK functionalized cationic liposomes (CPK-liposomes) and non-functionalized liposomes were prepared by the dehydration-rehydration method as described elsewhere (18). In short, lipids (DSPC, DOTAP and cholesterol in a 2:1:1 molar ratio, optionally including 1 mol% CPK) were mixed and the organic solvent was evaporated using a rotary evaporator. The lipid film was hydrated in 10 mmol/L HEPES, 280 mmol/L sucrose buffer in the presence of glass beads and lyophilized overnight. The resulting lipid cake was rehydrated with filtered Milli-Q® water to a final volume of 2 ml and homogenized using a LIPEX extruder (Evonik, Canada) over a stacked 400 nm & 200 nm Whatman® Nuclepore Track-Etch membrane (GE Healthcare, Chicago, IL, United States). Figure 1 shows a schematic design of the different types of liposomes used in this study. The Bet v 1 containing liposomes were prepared by adding 50 µg of Bet v 1 (as was determined by BCA)) to a 1 mg/ml liposome suspension. The pepE-Bet v 1 containing liposomes contained an equal amount of Bet v 1. The mixtures were incubated for at least 15 min before use.

### Liposome characterization

The hydrodynamic diameter (Z_ave_) and polydispersity (PDI) of liposome formulations and of alum-adsorbed Bet v 1 were measured by dynamic light scattering (DLS) using a Zetasizer Nano Zs (Malvern Instruments Ltd., Worcestershire, United States). The zeta potential is an electric field that exists between the surface of a particle and the surrounding liquid phase. It's an important physical characteristic of (nano)particles that affects their stability in a suspension and influences their interaction with cells (23). The zeta potential of all liposome formulations and alum-adsorbed Bet v 1 were measured using laser Doppler electrophoresis (lDe) on the same machine with a Zeta Dip Cell (Malvern Instruments Ltd.). Each sample was diluted 100-fold in 10 mmol/L HEPES buffer (pH 7.4, 0.2 µm filtered) before measurement.

### ImmunoCAP IgE inhibition

IgE binding-potency of pepE-Bet v 1-CC-CPK was determined by ImmunoCap IgE inhibition assay using rBet v 1 ImmunoCAPs (t215). The starting concentration of pepE-Bet v 1-CC-CPK and the pepE-Bet v 1 control were the same concentration used for the immunization study (50 µg/ml). The included Bet v 1 and pepE-Bet v 1 control samples started at 100 µg/ml and 50 µg/ml respectively. All samples were serially diluted (10-fold dilutions) in 10 mmol/L HEPES, 280 mmol/L sucrose, pH 7.4 and pre-incubated 1:1 (v/v) for 30 min at room temperature with a serum pool. The pool, composed of 36 birch pollen allergic patient reference sera from the CREATE project (24), was pre-diluted to approximately 14 kU/L of Bet v 1 -specific IgE prior to mixing (1:1 v/v) with diluted pepE-Bet v 1-CC-CPK liposomes and control samples. For the uninhibited value serum was incubated with the HEPES/sucrose buffer.

### Rat basophil leukemia (RBL) assay

To assess the allergenicity of pepE-Bet v 1-CC-CPK-liposomes, their ability to induce mediator release from effector cells was compared to that of soluble Bet v 1 and pepE-Bet v 1. For this, rat basophil leukemia cells (RBL-2H3), transfected with the human high-affinity IgE receptor (Fc*ε*RI), were sensitized with serum of Bet v 1 sensitized birch pollen allergic patients (24), and a *β*-hexosaminidase mediator release assay was performed as previously described (25). A detailed description of the method can be found in the supplementary Materials and Methods.

### Animal studies

Mice were immunized subcutaneously on day 0, 7 and 14 with pepE-Bet v 1-CC-CPK-liposomes or alum-adsorbed Bet v 1 (1 mg alum per dose). The groups administered with Bet v 1 containing preparations received the equivalent of 10 µg Bet v 1 per dose (200 µl). Control groups received either buffer (10 mmol/L HEPES, 280 mmol/L sucrose, pH 7.4), pepE-Bet v 1, CPK-liposomes, pepE-Bet v 1 liposomes or Bet v 1 CPK-liposomes. Serum for antibody detection was collected on days −1, 6, 13 and 20. On day 27, 28 and 29 the animals received an intranasal challenge under 3% (v/v) isoflurane anesthesia with 30 µl 100 µg/ml birch pollen extract (BPE) in PBS to further boost the immune response. On day 31, the mice were sacrificed and blood and lung draining lymph nodes were collected to analyze Bet v 1 specific levels of IgG_1_, IgG_2a_ and IgE in serum and to determine the production of cytokines (IL-4, IL-5, IL-13, IL-10 and IFN-*γ*) upon Bet v 1-specific stimulation of cells from lung-draining lymph nodes.

### Determination of bet v 1 specific antibodies

Serum was analyzed for the level of Bet v 1-specific immunoglobulins by ELISA (IgE and IgG_1_: Opteia, BD, San Diego, CA, United States, IgG_2a_: eBioscience) as previously described (26). For Bet v 1-specific IgG_1_ and IgG_2a_, Maxisorp plates were coated overnight with Bet v 1 and with purified anti-mouse IgG_1_ and anti-mouse IgG_2a_ for the standards. After blocking with FCS (10%), serum samples (IgG_1_: 10,000-fold diluted, IgG_2a_: 250-fold diluted) were incubated for 2 h and followed by an HRP-conjugated anti-IgG_1_ or anti-IgG_2a_ detection step, according to the manufacturer's instructions. For Bet v 1-specific IgE detection the plates were coated with an anti-IgE capture antibody, according to manufacturer's instructions. Subsequently, the wells were incubated with biotinylated Bet v 1 followed by streptavidin-horseradish peroxidase conjugate incubation to detect Bet v 1-specific IgE. Murine IgE, IgG_1_ and IgG_2a_ were used as standards to quantify the immunoglobulins.

### *Ex vivo* re-stimulation of lung draining lymph node cells

Lung draining lymph node cell suspensions were plated in 96-well round bottom plates at a density of 2 × 10^5^ cells per well in RPMI containing 50 µg/ml gentamicin, 5% (v/v) FCS and 50 µM 2-mercaptoethanol. The cells were re-stimulated for 4 days with 10 µg/ml Bet v 1. Expression levels of cytokines (IL-4, IL-5, IL-10, IL-13, and IFN-*γ*) were determined in the supernatant by ELISA (eBioscience).

### Statistics

Data was processed and statistically analyzed in GraphPad v9.1.0 (Prism) for Windows. The kinetic antibody levels (IgG_1_ and IgG_2a_) were analyzed with a two-way ANOVA and subsequent Tukey's multiple comparison test. Differences between group means at endpoints (IgE, cytokine levels, half-max *β*-hexosaminidase mediator release and antibody ratios) were compared with a one-way ANOVA and subsequent Tukey's multiple comparison test. *P* values < 0.05 were considered significant.

## Results

### Physicochemical characterization of CPK-liposomes before and after antigen adsorption

Characterization of purified CPK, peptide K_3_ and purification of the pepE-Bet v 1 fusion protein by affinity chromatography can be found in the supplements. Peptide K_3_ and CPK eluted after respectively 7.77 and 4.37 min during LC (Supplementary Figures S1 and S2). The molecular masses of peptide K_3_ and CPK were comparable to their theoretical values of respectively 2680.6 and 3747.2 Da. Based on its primary structure, the pepE-Bet v 1 fusion protein has a predicted molecular weight of 20.055,70 Da which was confirmed by MS (20.053 Da; data not shown). The Bet v 1 protein had a molecular mass of 17.429 Da as determined by MS (data not shown). Compared to Bet v 1 the pepE-Bet v 1 fusion protein showed a similar secondary structure as indicated by CD (Supplementary Figure S5).

PepE-Bet v 1 adsorption to CPK-liposomes *via* coiled coil formation increased the liposome size and polydispersity index (PDI), while the zeta potential decreased. The coupling efficiency between pepE-Bet v 1 and the CPK liposomes was determined by spinning down the liposomes in a vivaspin® column followed by measuring the amount of unbound pepE-Bet v 1 in the flow through by RP-HPLC. The coupling efficiency was typically >90% (data not shown). All liposome formulations had a hydrodynamic diameter of approximately 200 nm and a positive zeta potential (Table 1). The pepE-Bet v 1-CC-CPK-liposomes were slightly larger than the other formulations. The zeta potential was lower after coiled-coil mediated antigen adsorption. In contrast to the liposome formulations, alum-adsorbed Bet v 1 showed a slightly negative zeta potential and a hydrodynamic diameter >1000 nm.

**Table 1:** Overview of all formulations and their physicochemical characteristics (mean values ± SD, *n* = 3-5). All formulations contained the same buffer composed of 10 mmol/L HEPES, 280 mmol/L sucrose, pH 7.4.

Formulation	Antigen/carrier ratio (w/w)	Hydrodynamic diameter (nm)	PDI^†^	Zeta potential (mV)
Liposomes (non-functionalized)	n.a.^‡^	179.3 ± 13.8	0.073 ± 0.039	48.1 ± 3.3
CPK-liposomes	n.a.	176.7 ± 14.4	0.071 ± 0.047	44.9 ± 6.2
PepE-Bet v 1-CC-CPK liposomes	1/10	207.2 ± 10.7	0.159 ± 0.064	29.4 ± 4.2
Bet v 1 CPK-liposomes	1/10	189.3 ± 17.6	0.165 ± 0.029	40.0 ± 1.7
PepE-Bet v 1 liposomes	1/10	176.8 ± 16.4	0.145 ± 0.043	38.1 ± 3.9
Alum-adsorbed Bet v 1	1/100	1245.1 ± 131.9	0.318 ± 0.036	−6.3 ± 1.0

^‡^ n.a., not applicable.

^†^ PDI, polydispersity index.

### PepE-Bet v 1-cc-CPK-liposomes show hypo-allergenic IgE reactivity

**Figure 2:**
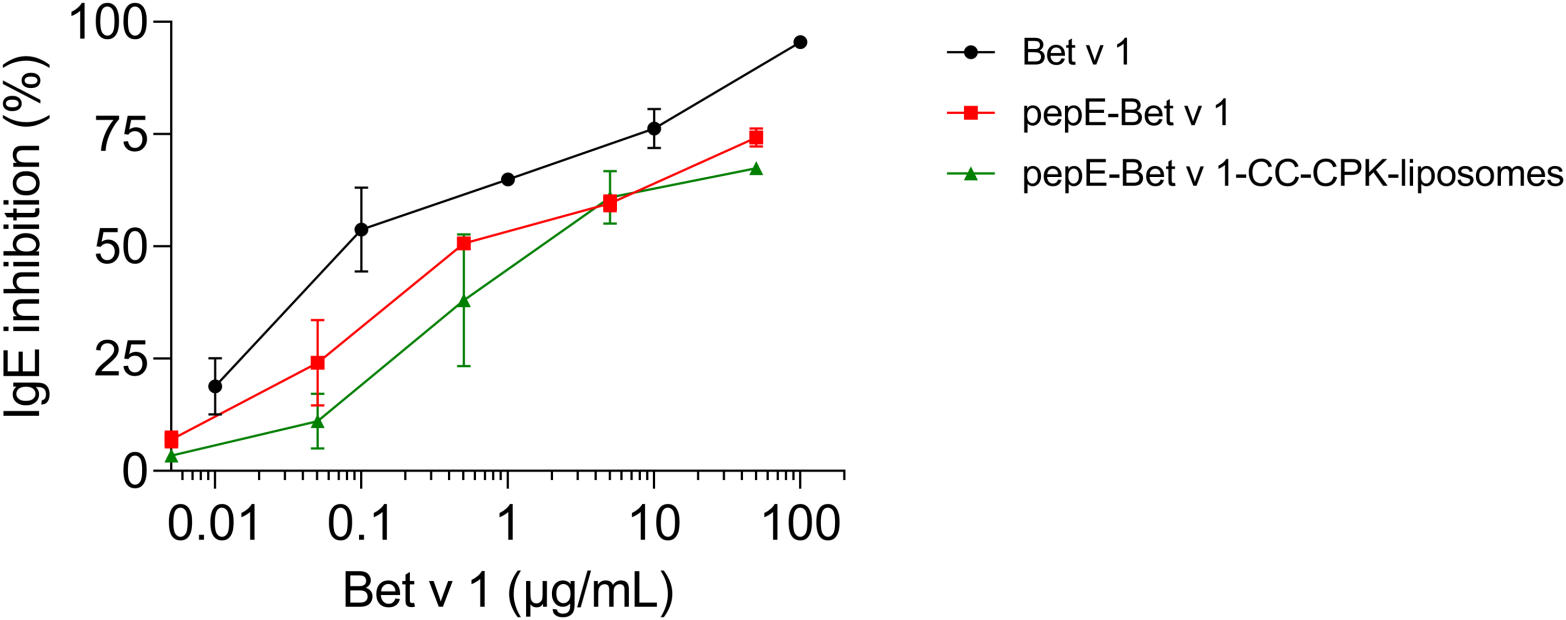
ImmunoCAP IgE inhibition assay. Serially diluted (10-fold) Bet v 1, pepE-Bet v 1 and pepE-Bet v 1-CC-CPK-liposomes were pre-incubated with pooled sera from birch pollen-allergic patients. After pre-incubation, the sample dilutions were loaded onto rBet v 1 ImmunoCAPs to determine the amount of uncomplexed IgE. An ImmunoCAP loaded with serum only was used as uninhibited IgE binding control. The shown graphs are normalized with regard to the uninhibited control and are an average of two independent experiments (mean ± SEM).

**Figure 3:**
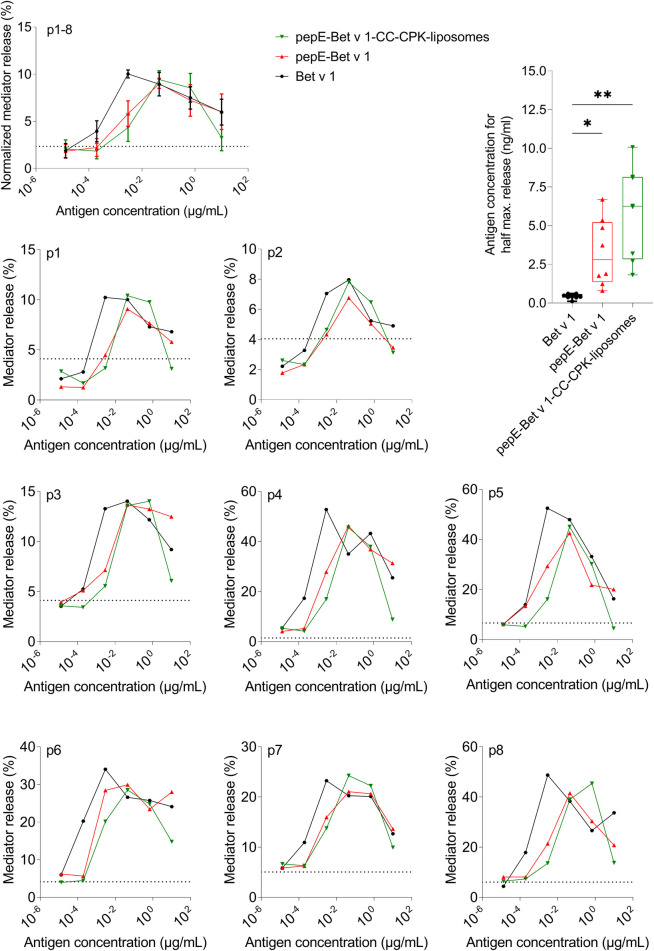
Allergenicity assessment by rat basophil leukemia cell assay. Rat basophils loaded with IgE from serum of Bet v 1-sensitized subjects were stimulated with different allergen formulations. Allergenicity was quantified by determining the concentration needed for half maximum mediator release. Differences between the groups were analyzed with a one-way ANOVA followed by Tukey's multiple comparisons test. Dotted line represents spontaneous mediator release by non-stimulated cells. The averaged, normalized curves of 8 patient sera (p1-8) as well as the patient specific mediator release curves showed a shift to the right of all pepE-Bet v 1 containing samples, indicating hypo-allergenicity. * = *p* < 0.05, ** = *p* < 0.01.

Next, we characterized the IgE binding capacity of the pepE-Bet v 1-CC-CPK-liposomes by testing their potency to inhibit IgE binding to rBet v 1 ImmunoCAPs. Compared to Bet v 1 the IgE inhibition curves of pepE-Bet v 1 and pepE-Bet v 1-CC-CPK-liposomes were shifted to approximately 4- to10-fold higher inhibitor concentrations indicative of a reduced IgE binding capacity (Figure 2). Subsequently, we tested the IgE cross-linking capacity of pepE-Bet v 1-CC-CPK-liposomes by RBL mediator release assay using human sera for passive sensitization. PepE-Bet v 1 and PepE-Bet v 1-CC-CPK-liposomes showed significant 8- and 14-fold higher concentrations to reach half maximum mediator release compared to soluble Bet v 1. Thus, pepE-Bet v 1 was already less allergenic than soluble Bet v 1 but association with either liposomes or CPK-liposomes lowered the IgE cross-linking capacity even more (Figure 3). The control groups are shown in supplementary Supplementary Figure S4.

### Coiled coil conjugation of bet v 1 triggers strong antibody responses in naïve mice

**Figure 4:**
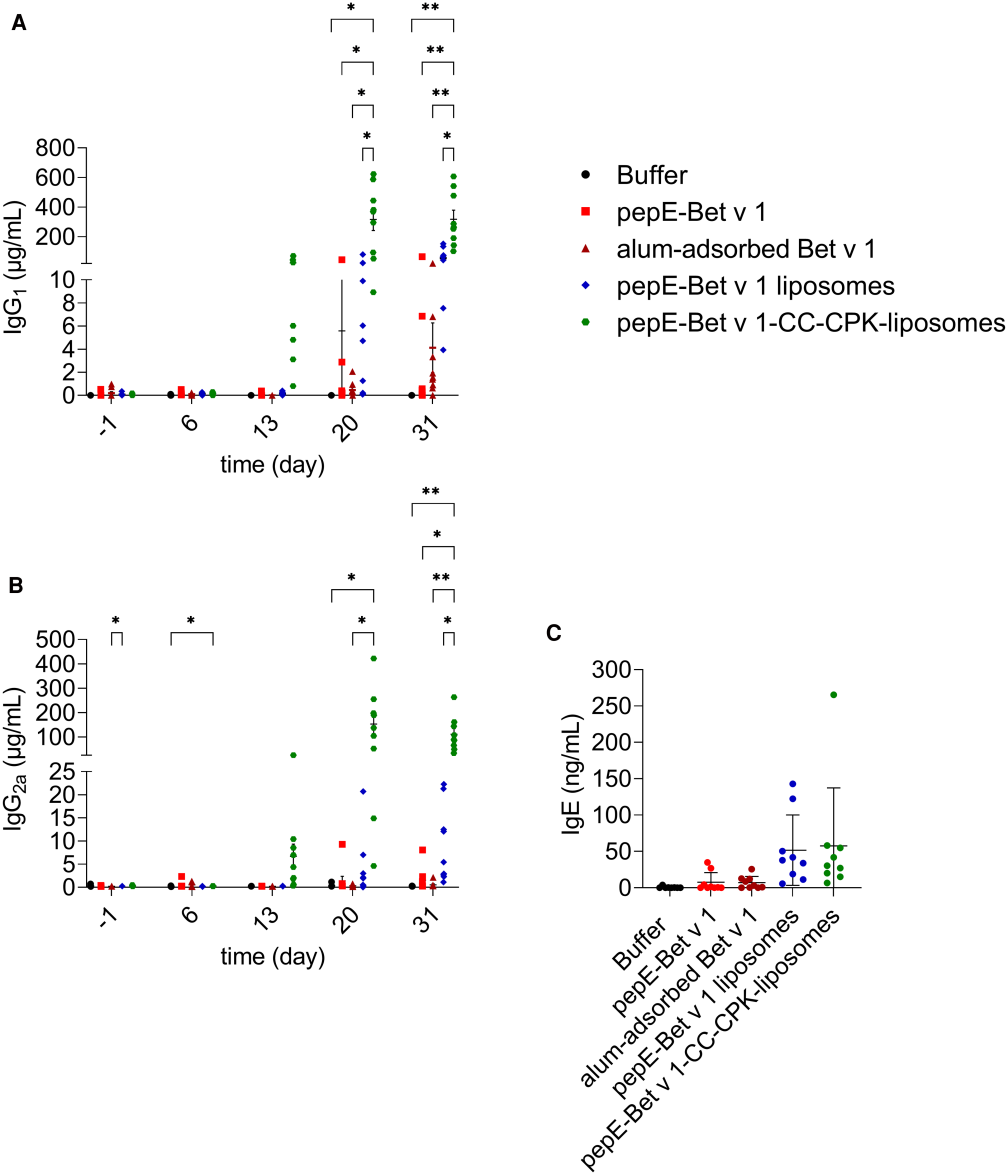
Murine immunogenicity. Mice (*n* = 9) were immunized with various formulations on day 0, 7 and 14 and received an intranasal challenge on day 27, 28 and 29 with birch pollen extract prior to the sacrifice (day 31). Bet v 1-specific IgG_1_ (**A**), IgG_2a_ (**B**) and IgE (**C**) were measured in serum collected at day −1, 6, 13, 20 and 31. Group mean differences were analyzed with two-way ANOVA (IgG) or one-way ANOVA (IgE) followed by Tukey's multiple comparisons test. * = *p* < 0.05, ** = *p* < 0.01.

To evaluate the immune response of pepE-Bet v 1-CC-CPK-liposomes, naïve mice were immunized 3 times at weekly intervals followed by intranasal birch pollen extract challenge. PepE-Bet v 1-CC-CPK-liposomes was compared to buffer, alum-adsorbed Bet v 1, and to pepE-Bet v 1 in solution and adsorbed to liposomes (without CC). All active treatment groups received an equimolar amount of Bet v 1. Mice that received pepE-Bet v 1-CC-CPK-liposomes had 77-fold higher IgG_1_ and 220-fold higher IgG_2a_ levels (Figures 4A,B, respectively) than alum-adsorbed Bet v 1 at the endpoint. Compared to soluble pepE-Bet v 1 and pepE-Bet v 1 liposomes these differences were, 31-fold and 5.6-fold (IgG_1_), and 73-fold and 12-fold (IgG_2a_), respectively. Moreover, only for the CC-conjugated Bet v 1, the induction of IgG antibodies was already detectable after the second injection. IgE levels were slightly higher upon immunization with pepE-Bet v 1-CC-CPK-liposomes (and other liposome preparations) than observed for soluble pepE-Bet v 1 and alum-adsorbed Bet v 1, but this did not reach significance (Figure 4C, Supplementary Figure S6C). In fact, the IgG_1_/IgE and IgG_2a_/IgE ratios in the pepE-Bet v 1-CC-CPK-liposomes group were significantly higher compared to alum-adsorbed Bet v 1 (IgG_1_: 11.2 vs. 1.72 and IgG_2a_: 3.78 vs. 0.25, Supplementary Figure S7). The other control groups are shown in Supplementary Figure S6.

### PepE-Bet v 1-cc-CPK-liposomes induce a strong mixed Th1/Th2/treg immune response

**Figure 5:**
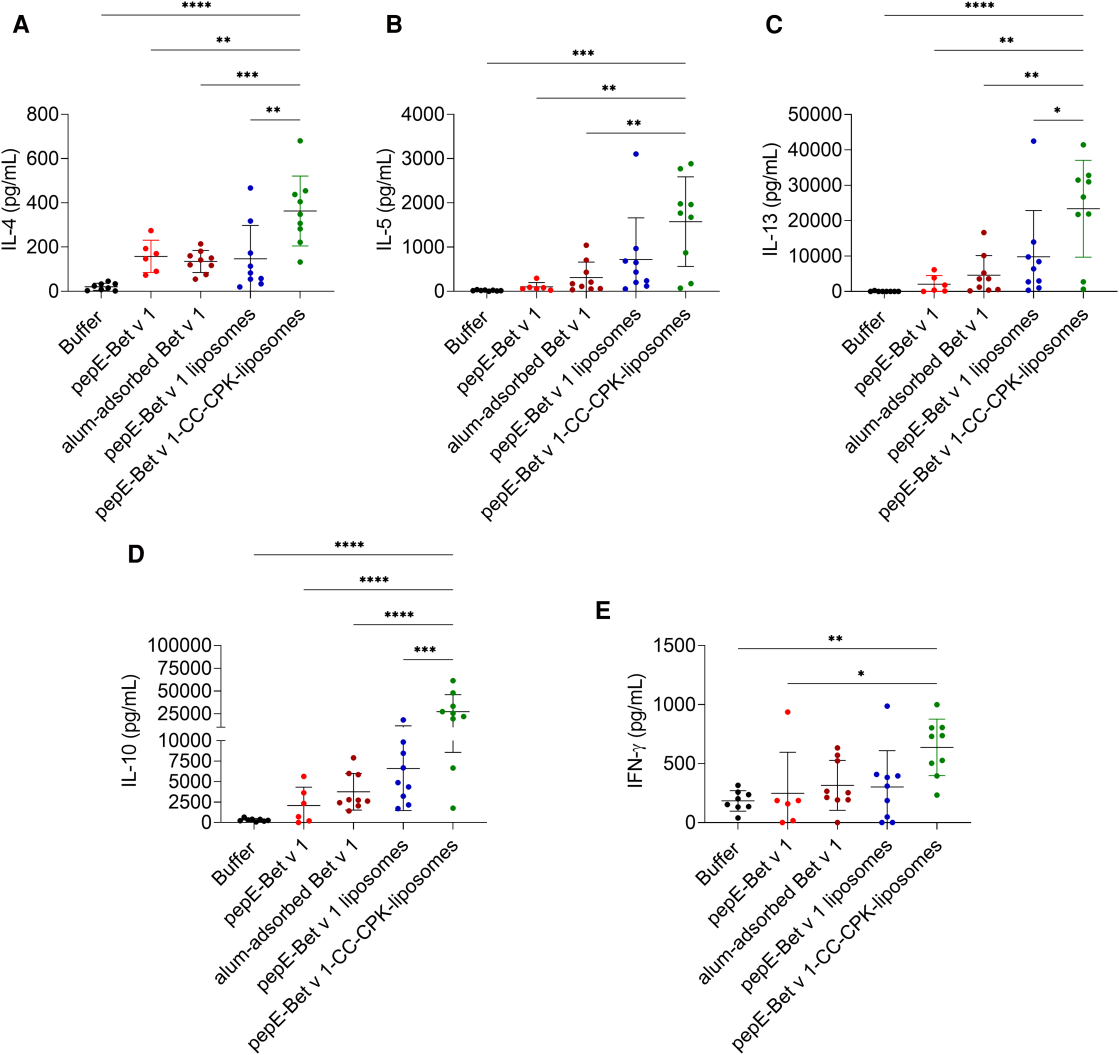
IL-4 (**A**), IL-5 (**B**), IL-13 (**C**), IL-10 (**D**) and IFN-*γ* (**E**) levels in supernatants of lung draining lymph node cells after *ex vivo* stimulation with Bet v 1. Bars represent the mean cytokine concentration and data points represent the signals for each individual mouse (*n* = 6-9). Group mean differences were analyzed with one-way ANOVA followed by Tukey's multiple comparisons test. * = *p* < 0.05, ** = *p* < 0.01, *** = *p* < 0.001, **** = *p* < 0.0001.

We also analyzed cytokine responses upon re-stimulation of lymph node cells (Figure 5). Compared to alum-adsorbed Bet v 1, pepE-Bet v 1-CC-CPK-liposomes induced 2.7-, 5.0-, 5.1-, 7.3- and 2.0-fold higher levels of IL-4, IL-5 and IL-13 (Th2), of IL-10 (Treg) and of IFN-*γ* (Th1) respectively, i.e., a strong mixed Th1/Th2/Treg response. This is further illustrated by a significantly higher IL-10/IL-4 ratio of pepE-Bet v 1-CC-CPK-liposomes compared to alum-adsorbed Bet v 1 (Supplementary Figure S9). The other control groups are shown in Supplementary Figure S8.

## Discussion

Previously we described the functionalization of cationic liposomes with a CC-forming peptide, which enhanced the immune response to model antigen OVA323 (18). In the current study, the CC functionalized cationic liposomes were used to design a new birch pollen SCIT formulation. In this design, pepE fused to the Bet v 1 N-terminus allowed CC-facilitated conjugation of the fusion protein to the functionalized liposome surface. Our results showed that pepE attachment to Bet v 1 interfered with IgE binding as well as IgE cross-linking, i.e., promoted hypo-allergenicity. Multivalent display of pepE-Bet v 1 on liposomes contributed to further increase of hypo-allergenicity. The phenomenon of hypo-allergenicity of allergen molecules upon surface-exposure on nanoparticles has also been reported by others. For example, despite being recognized by IgE, intact Fel d 1 displayed on the surface of virus-like particles failed to efficiently cross-link IgE on mast cells (27, 28). Similarly, trimeric Der *p* 2 displayed at high density on plant-produced bioparticles also showed reduced IgE cross-linking potential (29). How high-density surface expression translates into hypo-allergenicity is not completely clear. The tight interaction between allergens and the particle surface could sterically mask IgE epitopes from IgE binding, thereby preventing efficient IgE cross-linking and downstream induced mediator release (29). Alternatively, combined with a lower diffusion coefficient, the effective free allergen concentration of allergen bearing nanoparticles is strongly reduced which provides another physical explanation for the reduced IgE cross-linking capacity (28). Nevertheless, these promising results should be corroborated by more studies designed to elucidate how allergen-bearing nanoparticles reduce IgE cross-linking capacity compared to soluble allergen.

Noticeably, alum-adsorbed Bet v 1 induced weak humoral responses using the subcutaneous immunization route. We have confirmed these results in several other immunization experiments. However, using the same protocol, materials, reagents and amount of allergen we found strong humoral (and T-cell) responses *via* the intraperitoneal administration route. This protocol is based on the sensitization protocol that we used in a previously published study (26). Our results (29)suggest that in our case the low humoral response may have been at least partly due to the chosen administration route (30). Moreover, in all experiments, IgG induction was only detected at the last time point. Therefore, the used protocol may have been too short for alum-adsorbed Bet v 1 to detect the peak of its immune response but sufficient to detect the early and strong humoral response of our pepE-Bet v 1-CC-CPK-liposomes. Our results are also in line with other murine studies that used subcutaneously administered alum-adsorbed Bet v 1, showing a relatively slow and variable induction of IgG antibodies which were mostly observed after the immunizations and not in between (31, 32).

Interestingly, all tested liposome preparations in this study were much more potent antibody inducers compared to alum-adsorbed Bet v 1, with coiled-coil liposomes clearly being superior, both quantitatively and kinetically (earlier induction). Possibly, CC formation between pepE-Bet v 1 and the liposome leads to a stable, multivalent display on the particle surface which has been associated with efficient B-cell receptor cross-linking and subsequent antibody production (33). This could explain the adjuvant potency of cationic liposomes (16, 34) and the added value of CC attachment as opposed to weaker allergen adsorption methods to induce strong antibody responses.

Although murine models can provide a lot of insight, direct translation to humans is often difficult. For example, sensitization of naïve mice to allergens often leads to relatively strong IgG_1_ responses while IgE responses are usually quite low and sometimes undetectable. In atopic humans, the situation is commonly reversed: The relative strong IgE response to allergens is usually accompanied with low IgG responses (35). Humoral responses also differ between mice and humans in the therapeutic setting. In mouse models of AIT, two IgG isotypes, IgG_1_ and IgG_2a_, have been associated with relief of allergic symptoms (26, 27). In humans, allergic symptom relief after SCIT is correlated with increased levels of allergen-specific IgG_4_ (5, 36, 37). IgG_4_ lacks the typical pro-inflammatory properties of the other human IgG isotypes and is capable of blocking IgE binding to allergens, thereby reducing IgE-mediated effector cell stimulation, clinical symptoms as well as IgE-facilitated antigen presentation (38). Murine IgG_1_ and human IgG_4_ are considered equivalent and could exert a similar blocking effect (39) In contrast to human IgG_4_, murine IgG_1_ antibodies also have anaphylactic properties (40). Our coiled-coil liposomes induced both a strong IgG_1_ and IgG_2a_ antibody response against Bet v 1, suggesting a strong potential to induce blocking antibodies. The next step would be to study if these strong antibody responses are also induced in a mouse model of birch pollen SCIT and whether these responses associate with symptom reduction and outperform those induced by alum-adsorbed Bet v 1. Keeping the ambivalent role of mouse IgG_1_ in mind, it may be better to study this in a humanized mouse model (41).

The pepE-Bet v 1-CC-CPK-liposomes induced a strong but mixed Th1/Th2/Treg response compared to the Th2 biased response of alum-adsorbed Bet v 1. Although induction of Th2 cytokines could be undesirable for SCIT, human B cells require both IL-10 and IL-4 to switch to IgG_4_ production (35). In mice, the Th2 response induced by alum-adsorbed allergens is often used to induce allergic sensitization in SCIT models (30, 31, 42). Despite this pro-allergic potential of alum in animal studies, human clinical studies have shown that alum is capable of inducing regulatory responses and symptom relief after frequent administrations (26, 43–45). Therefore, the strong Th2 response and strong IL-10 induction of our pepE-Bet v 1-CC-CPK-liposomes could be interesting properties to achieve earlier and stronger protective immune responses during SCIT in humans.

The relatively high IL-10 expression levels induced by pepE-Bet v 1-CC-CPK-liposomes suggests a strong regulatory component. However, it is not clear which cells produced these large amounts of IL-10. In BALB/c mice IL-10 expression has been associated with Th2 immunity (46) Interestingly, mice immunized with the same CPK-liposomes bearing a pepE and OVA derived epitope conjugate showed induction of a CD4^+^ T-cell subset co-expressing IFN-*γ* and IL-10 (18). For this reason, it cannot be ruled out that at least part of the IL-10 levels observed after pepE-Bet v 1-CC-CPK-liposomes immunization was produced by similar Th1 skewed T-cells expressing both IFN-*γ* and IL-10.

In humans, induction of IL-10 producing regulatory T-cells capable of suppressing pro-inflammatory, type 2 T- and B-cell responses have been associated with improved clinical outcome during SCIT (8, 45). To bridge the gap between mice and humans, human based T-cell polarization studies using PBMCs or dendritic cells co-cultured with T-cells could shed more light on whether our pepE-Bet v 1-CC-CPK-liposomes are also capable of inducing similar allergen-specific IL-10 producing Tregs that are required for effective SCIT.

In summary, we have developed a novel SCIT candidate using functionalized, cationic liposomes surface-expressing Bet v 1 *via* CC formation. Their hypoallergenic character, strong humoral as well as mixed T-cell responses with a strong regulatory component induced by these nanoparticles are promising properties to design an alum-free birch pollen SCIT candidate.

## Data Availability

The original contributions presented in the study are included in the article/**Supplementary Material**, further inquiries can be directed to the corresponding author/s.
