# Supplementary material for: Cationic liposomes bearing Bet v 1 by coiled coil-formation are hypo-allergenic and induce strong immunogenicity in mice
Source: Front Allergy. 2023 Jan 10;3:1092262. doi: 10.3389/falgy.2022.1092262 (PMC9872006; doi:10.3389/falgy.2022.1092262)
Supplement: Supplementary file 1 [file Datasheet1.docx]

Supplementary Material

# Supplementary Materials and Methods

## Design, expression and purification of pepE-Bet v 1.

The gene of pepE-Bet v 1 was optimized for expression in E. coli and ligated into the p19b expression vector by GenScript (Piscataway, NJ, USA):

ATGTATGGAGAAATCGCAGCCCTTGAAAAAGAGATTGCCGCCTTAGAAAAAGAAATTGCCGCACTGGAAAAGGGTGTTTTCAATTACGAAACTGAGACCACCTCTGTTATCCCAGCAGCTCGACTGTTCAAGGCCTTTATCCTTGATGGCGATAATCTCTTTCCAAAGGTTGCACCCCAAGCCATTAGCAGTTTGAAAACATTGAAGGAAATGGAGGGCCTGGAACCATTAAGAAGATCAGCTTTCCCGAAGGCTTCCCTTTCAAGTACGTGAAGGACAGAGTTGATGAGGTGGACCACACAAACTTCAAATACAATTACAGCGTGATCGAGGGCGGTCCCATAGGCGACACATTGGAGAAGATCTCCAACGAGATAAAGATAGTGGCAACCCCTGATGGAGGATCCATCTTGAAGATCAGCAACAAGTACCACACCAAAGGTGACCATGAGGTGAAGGCAGAGCAGGTTAAGGCAAGTAAAGAAATGGGCGAGACACTTTTGAGGGCCGTTGAGAGCTACCTCTTGGCACACTCCGATGCCTACAACTAA. The underscored base pairs denote the DNA sequence coding for EIAALEKEIAALEKEIAALEK, the pepE amino acid sequence.

The pepE-Bet v 1 fusion protein was expressed in competent Novagen® E. coli BL21 (DE3) cells (Merck, Darmstadt, Germany) by transfecting the cells with 5 ng DNA followed by heat shock treatment at 42 °C. Subsequently, the transformed cells were grown on antibiotic selective LB plates containing ampicillin (50 µg/mL). Protein expression was induced in 5 mL DifcoTM Terrific Broth (BD Biosciences, Franklin Lakes, NJ, USA) cultures with 1 mmol/L IPTG at OD600 nm 0.6 for 3 hours at 37 °C. The highest producing clones were used for protein production by inoculating 4 L TB medium in a 5 L stirred tank coupled to a BIOSTAT® controller (Sartorius Stedim Biotech, Göttingen, Germany). Protein production was induced for 3 hours with 1 mmol/L IPTG when the cells reached an OD 600 nm of 0.6. Finally, the cells were pelleted by centrifugation at 4600 rpm and stored at -20 °C until further use. Frozen cell pellets were thawed on ice and re-suspended in ice cold lysis buffer (100 mmol/L sodium phosphate, 100 µg/mL lysozyme, pH 7.0) for 1 hour. The cells were disrupted by sonicating at 15 µm amplitude at 5 x 30 second bursts with 30 second intervals. Cellular debris was spun down at 20,000 g and 8°C for 20 minutes. The supernatant was collected. Bacterial DNA was precipitated with 0.4% (v/v) polyethyleneimine and spun down again at 12.000 g and 8 °C for 20 minutes. The supernatant was filtered through 0.2 µm before loading onto the affinity column.

An affinity purification column was prepared by coupling CGWG-(KIAALKE)3, to a supporting matrix. PepK was synthesized as described in the main article. The peptide was coupled to 6% cross-linked agarose beads functionalized with iodoacetyl groups (Sulfolink, Thermo Scientific) via the N-terminal cysteine according to the manufacturer’s instructions [23]. The affinity matrix was equilibrated with 5 column volumes (CV) 100 mmol/L sodium phosphate, 500 mmol/L sodium chloride, pH 7.0. Filtered supernatant was loaded onto the column and unbound proteins were washed out with 10 CV equilibration buffer. Bound pepE-Bet v 1 was eluted by lowering the pH with 5 CV of 100 mmol/L glycine HCl, pH 2.5 to unfold the pepE/pepK coiled coil. Elution fractions were collected and directly neutralized with (1:4, v/v) 1 mol/L TrisHCl, pH 9. Flow through, wash and elution fractions were analyzed with SDS PAGE (supplementary figure 2A and 2B). Elution fractions containing pepE-Bet v 1 were pooled and concentrated before gel filtration. The concentrate was loaded at 1 mL/minute onto a Superdex 75 pg column (GE Healthcare, Chicago, IL, US) equilibrated with 10 mmol/L HEPES, 280 mmol/L sucrose, pH 7.4 as a final step. Fractions containing the pure protein were pooled and stored at -20 °C until further use.

## SDS-PAGE

Purification fractions were analyzed with sodium dodecyl sulfate polyacrylamide gel electrophoresis (SDS-PAGE) under reducing conditions using 4-12% Bis-Tris gels (GE Healthcare) according to manufacturer’s instructions. Gels were stained with PageBlue Coomassie and de-stained overnight in water.

## Circular Dichroism

CD spectra were measured on a JASCO J-815 CD spectrometer connected to a Peltier temperature controller using a quartz cuvette with a path length of 1 mm. The reported spectra are averages of 10 spectra that were measured at 25 °C in quick succession, using a range of 190 to 260 nm with 1 nm intervals and a bandwidth of 1 nm. The protein concentrations were 14 µM.

## Rat basophil leukemia (RBL) assay

Per sample, 2 x 10^5^ transfected RBL-2H3 cells/well were passively sensitized overnight with sera derived from birch pollen allergic patients (n=8). The sera used in this study are reference sera from the CREATE project (ref). The sera were chosen either based on sIgE for Bet v 1 (ImmunoCAP^TM^) or sIgE RAST (birch), see table S1. To neutralize the complement system, the sera were incubated with P3X63Ag8.653 cells ((ATCC CRL-1580™), Manassas, VA, USA) prior to the sensitization step. For β-hexosaminidase release, the cells were stimulated with the samples in eight 15-fold dilution steps ranging from 10 µg/mL to 0.06 pg/mL Bet v 1 concentration. The cells were stimulated with the samples for one hour at 37 °C, 7% CO2 before the cell supernatant was incubated with the β-hexosaminidase substrate, 4-methyl umbelliferyl-N-acetyl-beta-D-glucosaminide, for another hour at 37 °C and then quenched with 0.2 M of glycine buffer (pH 10.7). The fluorescence was measured at an excitation and emission wavelength of 360 nm and 465 nm, respectively. The data are presented as percentage of cell release relative to the maximal enzyme release caused by cell lysis (10% Triton X-100, Sigma-Aldrich, Inc.), which was firstly corrected for spontaneous release (no serum sensitization). The normalization in the top graph was performed as follows. The results of all patients were normalized by calculating the ratio between the maximum Bet v 1 induced mediator release of patient 1 and the maximum mediator release values of all the other patients, resulting in a normalization factor for each patient serum. Per individual serum this factor was applied to normalize the data for each tested sample. The shown graph displays the mean of the normalized data for each sample. In figure S10, the mediator release at 10^-2^ and 10^-4^ µg/mL is shown for Bet v 1 and pepE-Bet v 1 before and after normalization.

# Supplementary Tables

**Table S1.** Overview of the birch pollen allergic patient sera used for the RBL assay.

| **Sera** | **Date of birch** | **Gender** | **total IgE  (kU/L)** | **birch pollen IgE (kU/L)** |
| --- | --- | --- | --- | --- |
| 01 | 6-6-1966 | male | 296 | 99.0 |
| 02 | 14-3-1960 | female | 307 | 77.8 |
| 03 | 24-3-1976 | female | 312 | 57.3 |
| 04 | 14-9-1969 | female | 336 | 62.2 |
| 05 | 15-9-1993 | female | 893 | 62.6 |
| 06 | NA | NA | NA | NA |
| 07 | NA | NA | NA | NA |
| 08 | 5-11-1968 | male | 819 | >100 |

# Supplementary Figures


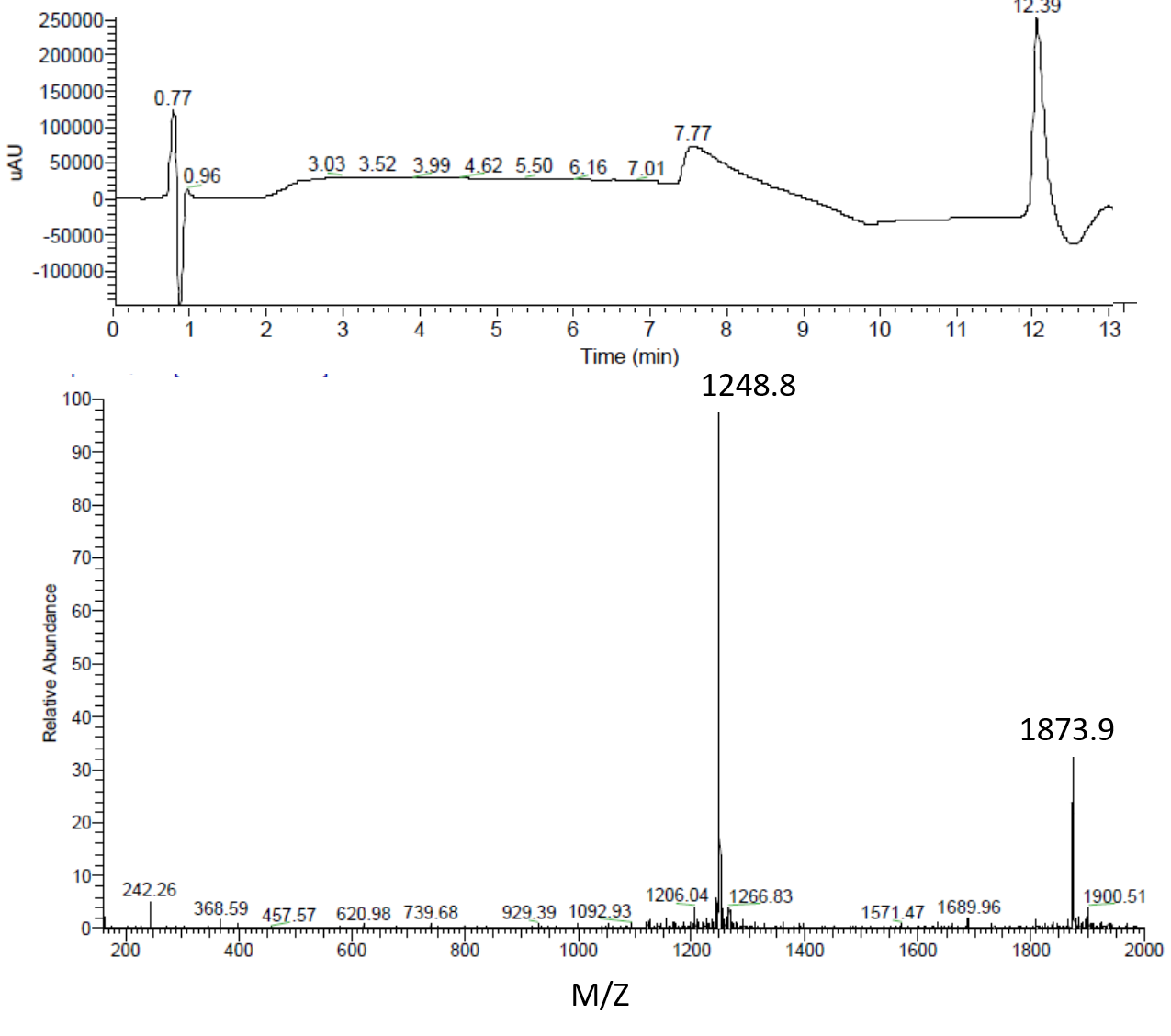


**Figure S1.** LC (top) chromatogram and MS (bottom) spectrum of the CPK peak eluting at 7.77 minutes. The peak at 12.39 minutes corresponded to the transition of solvents during the programmed gradient. The sequence of CPK is cholesterol-PEG4-(KIAALKE)_4_, which has a theoretical molecular mass of 3747.2. The expected m/z values are: 1874.6 and 1250.1 for 2+ and 3+, respectively.


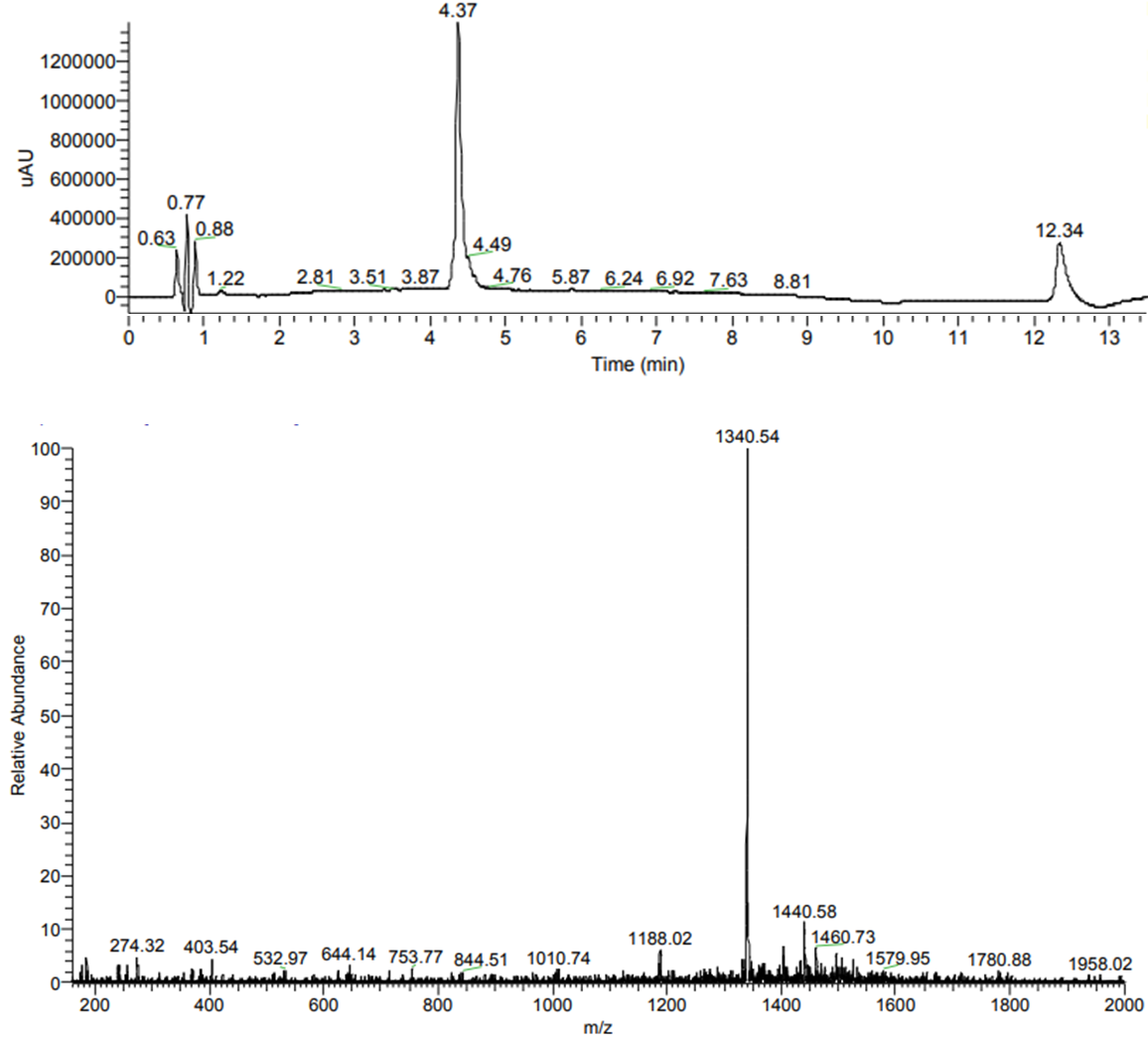


**Figure S2.** LC (top) chromatogram and MS (bottom) spectrum of peptide K_3_ (CGWG-(KIAALKE)_3_) eluting at 4.37 minutes. The peak at 12.34 minutes corresponded to the transition of solvents during the programmed gradient. The theoretical molecular mass is 2680.6 Da. The expected m/z values are 1341 and 895 for 2+ and 3+, respectively.


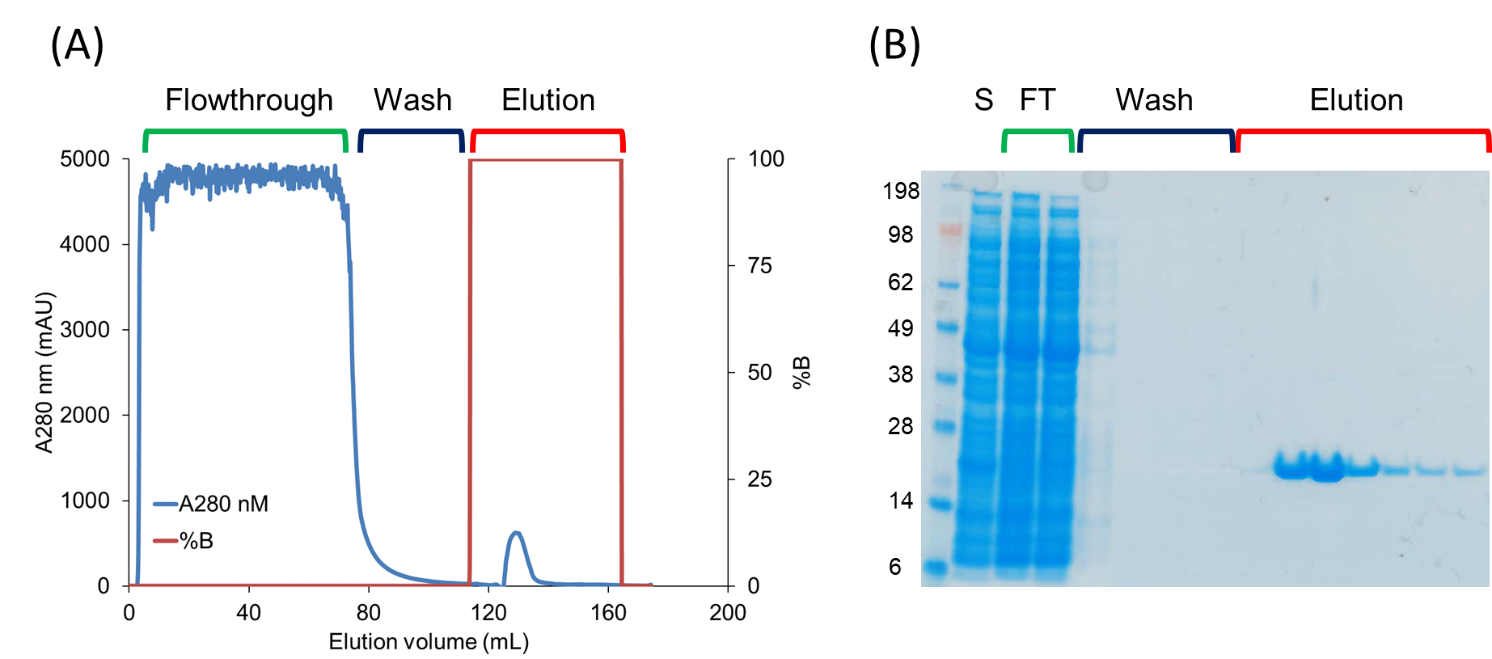


Figure S3. Affinity purification and analysis of pepE-Bet v 1. A) elution from pepK-affinity column shows one peak, in which only a protein with the correct molecular weight is found on SDS-PAGE under reducing conditions (B). S = Start material.

**Figure S4. Allergenicity assessment by rat basophil leukemia cell assay.** Rat basophils loaded with IgE from serum of Bet v 1-sensitized subjects were stimulated with different allergen formulations. Allergenicity was quantified by determining the concentration needed for half maximum mediator release. Differences between the groups were analyzed with a one-way ANOVA followed by Tukey’s multiple comparisons test. Dotted line represents spontaneous mediator release by non-stimulated cells. The averaged, normalized curves of 8 patient sera (p1-8) as well as the patient specific mediator release curves showed a shift to the right of all pepE-Bet v 1 containing samples, indicating hypo-allergenicity. * = p < 0.05, ** = p < 0.01.


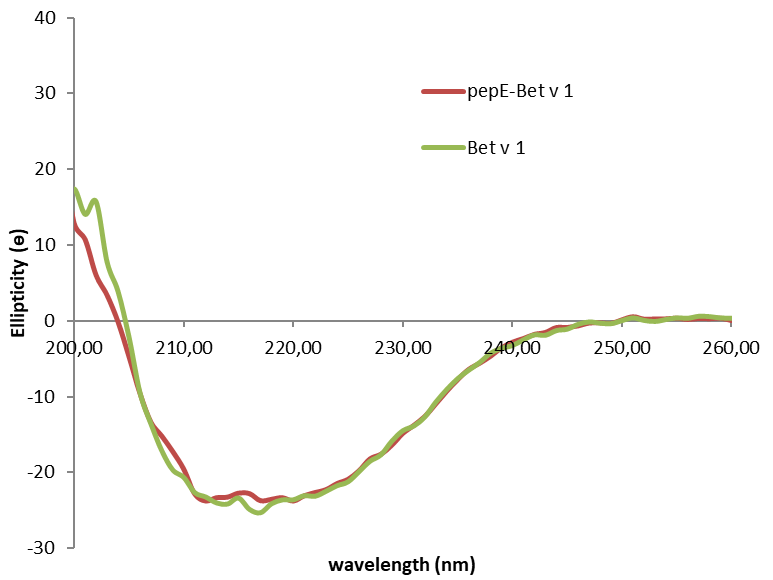


**Figure S5. Far UV CD spectrum of pepE-Bet v 1.** The secondary structure of pepE-Bet v 1 was comparable to Bet v 1 and was not affected by the conjugation of pepE.

**Figure S6.** **Murine immunogenicity.** Mice (n=9) were immunized with various formulations on day 0, 7 and 14 and received an intranasal challenge on day 27, 28 and 29 with birch pollen extract prior to the sacrifice (day 31). Bet v 1-specific IgG_1_ (A), IgG_2a_ (B) and IgE (C) were measured in serum collected at day -1, 6, 13, 20 and 31. Group mean differences were analyzed with two-way ANOVA (IgG) or one-way ANOVA (IgE) followed by Tukey’s multiple comparisons test. * = p < 0.05, ** = p < 0.01.

**Figure S7.** IgG_1_/IgE (left) and IgG_2a_/IgE right) ratios. Group means were compared with a one-way ANOVA and subsequent Tukey’s multiple comparison test. * = p < 0.05, ** = p < 0.01.

**Figure S8.** IL-4 (A), IL-5 (B), IL-13 (C), IL-10 (D) and IFN-γ (E) levels in supernatants of lung draining lymph node cells after *ex vivo* stimulation with Bet v 1. Bars represent the mean cytokine concentration and data points represent the signals for each individual mouse (n=6-9). Group mean differences were analyzed with one-way ANOVA followed by Tukey’s multiple comparisons test. * = p < 0.05, ** = p < 0.01, *** = p < 0.001, **** = p < 0.0001.

**Figure S9.** IL-10/IL-4 (A), IL-10/IL-5 (B), IL-10/IL-13 (C) and IL-10/IFN-γ (D) ratios. Bars represent the mean cytokine ratio and data points represent the cytokine ratio per mouse (n=6-9). Group mean differences were analyzed with one-way ANOVA followed by Tukey’s multiple comparisons test. * = p < 0.05, ** = p < 0.01.

**Figure S10.** Mediator release at 10^-2^ and 10^-4^ µg/mL. The graphs show the difference between Bet v 1 and pepE-Bet v 1 before (top row) and after normalization (bottom row). Group mean differences were analyzed with Student’s t-test. ** = p < 0.01, **** = p < 0.0001
